# Operative treatment of 2-part surgical neck fractures of the proximal humerus (AO 11-A3) in the elderly: Cement augmented locking plate Philos™ vs. proximal humerus nail MultiLoc®

**DOI:** 10.1186/s12891-016-1302-6

**Published:** 2016-10-28

**Authors:** Tobias Helfen, Georg Siebenbürger, Marcel Mayer, Wolfgang Böcker, Ben Ockert, Florian Haasters

**Affiliations:** Department of Trauma Surgery, Ludwig-Maximilians-University (LMU), Campus Innenstadt, Nußbaumstr. 20, D-80336 Munich, Germany

**Keywords:** Humeral head, Osteosynthesis, Plate, Nail, Osteoporosis

## Abstract

**Background:**

Proximal humeral fractures are with an incidence of 4–5 % the third most common fractures in the elderly. In 20 % of humeral fractures there is an indication for surgical treatment according to the modified Neer-Criteria. A secondary varus dislocation of the head fragment and cutting-out are the most common complications of angle stable locking plates in AO11-A3 fractures of the elderly. One possibility to increase the stability of the screw-bone-interface is the cement augmentation of the screw tips. A second is the use of a multiplanar angle stablentramedullary nail that might provide better biomechanical properties after fixation of 2-part-fractures. A comparison of these two treatment options augmented locking plate versus multiplanar angle stable locking nail in 2-part surgical neck fractures of the proximal humerus has not been carried out up to now.

**Methods/Design:**

Forty patients (female/male, ≥60 years or female postmenopausal) with a 2-part-fracture of the proximal humerus (AO type 11-A3) will be randomized to either to augmented plate fixation group (PhilosAugment) or to multiplanar intramedullary nail group (MultiLoc). Outcome parameters are Disabilities of the Shoulder, Arm and Hand-Score (DASH) Constant Score (CS), American Shoulder and Elbow Score (ASES), Oxford Shoulder Score (OSS), Range of motion (ROM) and Short Form 36 (SF-36) after 3 weeks, 6 weeks, 3 months, 6 months, 12 and 24 months.

**Discussion:**

Because of the lack of clinical studies that compare cement augmented locking plates with multiplanar humeral nail systems after 2-part surgical neck fractures of the proximal humerus, the decision of surgical method currently depends only on surgeons preference. Because only a randomized clinical trial (RCT) can sufficiently answer the question if one treatment option provides advantages compared to the other method we are planning to perform a RCT.

**Trial registration:**

Clinical Trial (NCT02609906), November 18, 2015, registered retrospectively.

## Background

Proximal humeral fractures are with an incidence of 4–5 % the third most common fractures in the elderly [1]. These are the second most common upper-limb fracture after distal radial fractures [2, 3]. Sixty-five percent of all patients with a proximal humeral fracture are older than 60 years [1]. Being aware of the demographic change there will even be an increase of incidence of these fractures. Kannus et al. showed an incidence of 298 per 100,000 in the at least 80 years old patients in 2007 [4]. Palvanen et al. predict an increase of incidence of 50 % until 2030 [1].

Approximately 80 % of all humeral fractures are minimally or non-displaced and can be treated conservatively with a good functional result [5]. In 20 % of humeral fractures there is an indication for surgical treatment according to the modified Neer-Criteria [6]. These criteria are fulfilled if there is an angulation of at least 45 ° between fracture fragments, a displacement of the humeral shaft against the humeral head of at least 1 cm or a dislocation of the greater tuberosity of at least 5 mm [7]. Up to now there is no evidence for superiority of any surgical treatment in literature [8]. At the moment the most frequently used surgical technique for treatment of proximal humeral fractures is the angle stable plate fixation. There are various publications concerning this topic published by our research group [9, 10]. In our 10-years results a majority of patients showed excellent and good, but also 16 % showed unsatisfactory results after locking plate fixation [10]. Main risk for poor outcome was revision surgery caused by secondary displacement (14 %) which is also confirmed by results of other studies [11]. In a further study our research group could show that there is a higher risk for secondary displacement in 2-part-fractures with a gross primary dislocation or a large metaphyseal fracture zone (AO 11-A3), especially in osteoporotic patients [12]. Moreover these are common fractures and because of that a problem in surgical treatment.

A secondary varus dislocation of the head fragment and cutting-out are the most common complications of angle stable locking plates in AO 11-A3 fractures of the elderly. The primary reason for this mechanism of failure is certain instability of transmetaphyseal fractures in the region of the surgical neck caused by loss of impaction in a porous spongiosa. Because of that the forces on the head screws are high while the so called screw-bone-interface is rather weak after a surgical treatment.

Currently there exist various approaches to avoid a failure of the primary screw implantation. One possibility to increase the stability of the screw-bone-interface is the cement augmentation of the screw tips [13–18]. To date there exists no clinical study that reports the results of locking plate fixation and the augmentation of cannulated head screws although it is a widely used method in everyday surgery, especially in the elderly.

A second possibility to prevent secondary displacement after surgical treatment of 2-part-fractures is the use of an intramedullary nails [19–22]. A further development of intramedullary nails is multiplanar nailing. Screws can be inserted in various different levels and directions which can lead to a clearly higher stability [23–25].

A comparison of these two treatment options augmented locking plate versus multiplanar angle stable locking nail in 2-part surgical neck fractures of the proximal humerus has not been carried out up to now.

### Aim of the study

This randomized clinical study aims to compare cement augmented locking plate fixation versus a multiplanar humeral nail system for the treatment of displaced 2-part proximal humeral fractures in the elderly patient in terms of complication rate, shoulder function, quality of life and patient satisfaction.

### Hypothesis

We anticipate the cement augmented angle stable plate fixation system Philos™ with augmentation (Depuy-Synthes) achieves significant better outcome concerning intra- and postoperative complication and revision rate, functional outcome and patient satisfaction compared with the multiplanar proximal MultiLoc®-Nail (Depuy-Synthes).

## Methods/Design

### Study design

For comparative evaluation of the two treatment options: cement augmented plate fixation versus multiplanar intramedullary locking nail in proximal humeral fractures (AO Type 11-A3) we planned a single-centre parallel group, randomized controlled trial (RCT), following the CONSORT statement guidelines [26].

Forty patients will be randomized to either to augmented plate fixation group (PhilosAugment) or to multiplanar intramedullary nail group (MultiLoc). Patients will be recruited at our hospital (level I trauma centre). All subjects must provide written informed consent.

### Population, screening and randomization

**Fig. 1:**
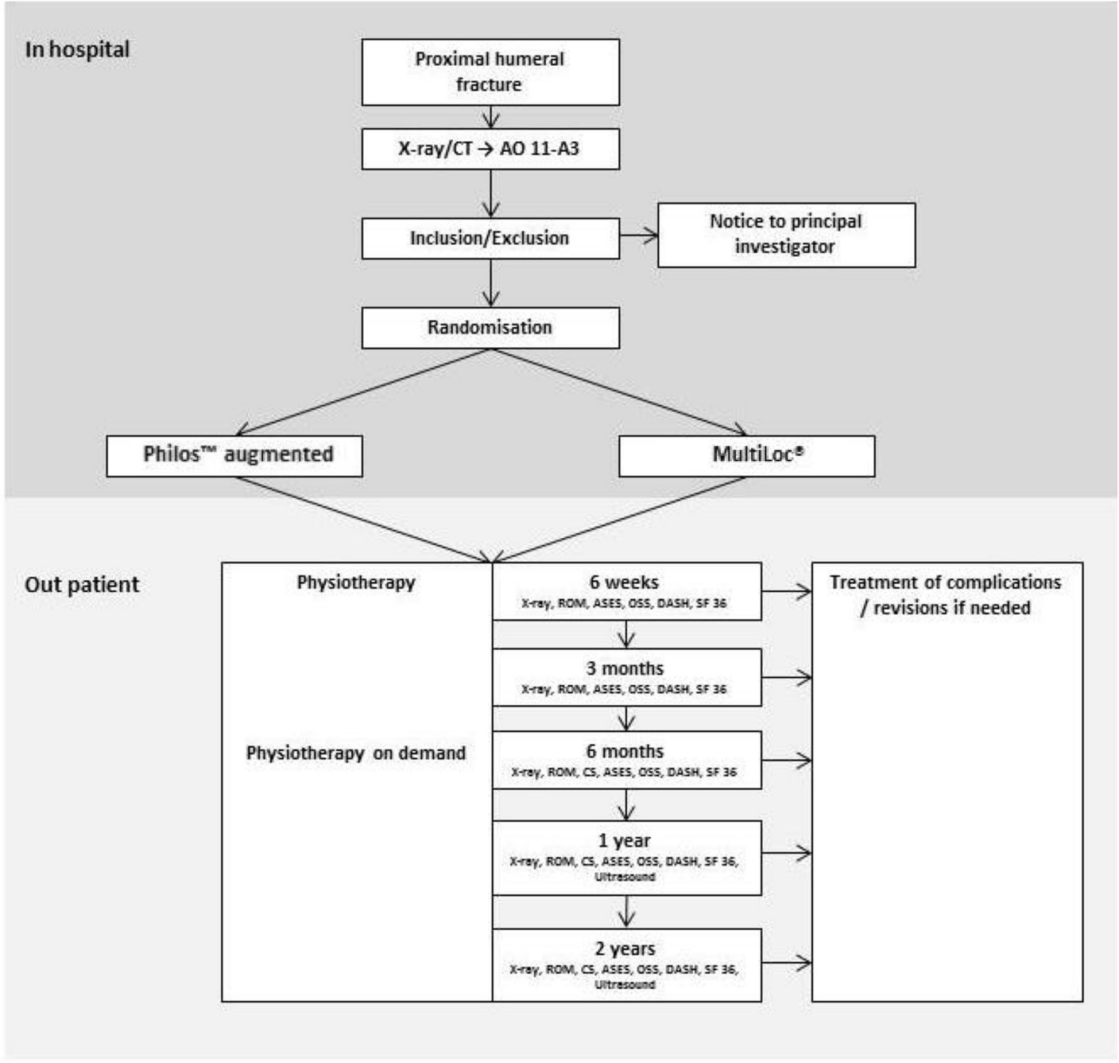
Randomization and follow-up workflow

The study will be conducted at the Department of Trauma Surgery, University of Munich (LMU), Germany. Patients will be screened within the regular emergency unit settings using the AO-Classification. Patients (female/male, ≥60 years or female postmenopausal) with a 2-part surgical neck fractures of the proximal humerus (AO type 11-A3). A correct fracture classification is ensured by CT-scans for all participants. Inclusion and exclusion criteria are listed in Table 1. Within 7 days, participants must be included. Inclusion and exclusion criteria are listed in Table 1. Within 7 days, participants must be included and randomized by sealed envelope (opaque, not resealable) drawing (Fig. 1). Each subject will have a unique identification number and keep that number throughout the study. Sequence generation was performed by online Statistical Computing Web Programme: www.randomization.com.

**Table 1:** Inclusion- and exclusion criteria

Inclusion criteria	Exclusion criteria
• Age: ≥60 years, or female postmenopausal • 2-part fracture according to AO-classification (Arbeitsgemeinschaft für Osteosynthesefragen): AO 11-A3 • Signed informed consent • Patient can read and understand German	• Refusal to participate in the study • Not Independent • Dementia and/or institutionalized • Does not understand written and spoken guidance German • Pathologic fracture or a previous fracture of the same proximal humerus • Alcoholism or drug addiction, e.g., in the emergency department, breathalyzer indicates blood alcohol concentration of more than 2 % • Other injury to the same upper limb requiring surgery • Major nerve injury (e.g., complete radial- or axillary nerve palsy) • Rotator cuff tear arthropathy • Open fracture • Multi-trauma or -fractured patient • Fracture dislocation or head-splitting fracture • Non-displaced fracture • Isolated fracture of the major or minor tubercle • Any medical condition that excludes surgical treatment • Pregnancy

### Interventions

All fractures will initially be immobilised by a Gilchrist-bandage. This is the same procedure as for patients who do not attend this or any other trial and will be performed by the doctor on-duty in the emergency room. Patients will be admitted to the trauma ward after that. On the same day or at the latest on day after this patients will be informed about the current investigation, screened, included and randomized by one of the study investigators to our trial after written consent will be obtained as described above. The patients are allowed to make their decision within 24 h.

Operative treatment is exclusively performed by the below mentioned study investigators. The intervention group will be treated by the angle stable 3-hole plate fixation system Philos™ with augmentation (Depuy-Synthes) the comparison group by the multiplanar proximal humeral nail MultiLoc® with the length of 160 mm and a diameter according to the humeral shaft ((Depuy-Synthes). For plate fixation screws are set in a standardized fashion: Seven screws placed subcortical in row A, B, D and F as well as 3 screws bicortical in the shaft (one proximal non-locking and two distal locking screws). Whenever possible (according to contrast dye testing) the screws in row A and E are augmented with 0.5 ml Traumacem V+ (Depuy-Synthes). Alternatively screws in row B and D are used. The MultiLok nail is standardized fixed with three 4.5 mm MultiLoc screws at levels A, B and D. Two additional 3.5 mm screw-in-screws are used at levels A and B and aimed posteriorly. In the shaft one ascending calcar screw and two multiplanar distal locking screws are implanted. All study investigators are very experienced with both devices and have completed their learning curves.

### Types of outcomes and follow-up

The primary outcome will be the Disabilities of the Shoulder, Arm and Hand (DASH) Score after 24 months. The secondary outcome parameters are listed in Table 2 (Disabilities of the Shoulder, Arm and Hand-Score (DASH), Constant Score (CS), American Shoulder and Elbow Score (ASES), Oxford Shoulder Score (OSS), Range of motion (ROM) and Short Form 36 (SF-36) after 3 weeks, 6 weeks, 3 months, 6 months, 12 and 24 months. Further evaluation will be: Fracture reduction and healing results (radiographic findings), complications (Table 3) and revisions. Complications will be classified as described in Table 2. Follow-up examinations will take place after 3 weeks, 6 weeks, 3 months, 6 months, 12 and 24 months (Fig. 1). There will be no blinding of outcome assessment (Table 2).

**Table 2:** Assessment and procedure of the trial

Assessment	Pre-operative	1. visit 6 weeks	2. visit 3 months	3. visit 6 months	4. visit 12 months	5. visit 24 months
Doctor’s visit	X	X	X	X	X	X
x-ray (true a.p., lateral, axial view)	X	X	X	X	X	X
CT-scan	X					
Inclusion criteria	X					
Consent	X					
Medical history	X					
ROM		X	X	X	X	X
CS			X	X	X	X
ASES		X	X	X	X	X
OSS		X	X	X	X	X
DASH		X	X	X	X	X
SF-36		X	X	X	X	X
Ultrasound for rotator cuff status					X	X

*ROM* range of motion, *CS* Constant Score, *ASES* American Shoulder and Elbow Score, *OSS* Oxford Shoulder Score, *DASH* Disabilities of the Shoulder, Arm and Hand, *SF-36* Score and Short Form 36

**Table 3:** Complications

Classification of complications	
A) Implant associated complications/revisions	
A1) varus displacement	
A2) dorsal displacemenmt	
A3) varus and dorsal displacement	
A1-3) + S additional screw-cut-out	
A4) humeral shaft perforation	
A5) implant breaking	
A6) subacromial impingement	
A7) intraarticular cement malposition	
B) Non-implant associated complications/revisions	
B1) frozen shoulder	
B2) hematoma/seroma	
B3) infection	
B4) impairment of wound healing	
B5) nerve injury	
B6) avascular necrosis	
C) Technical complications/revisions	
C1) malposition of the implant	
C2) inadequate reduction	
D) Medical complications	
D1) venous thrombembolisms	
D2) pulmonary complications	
D3) cardiac complications	

### Sample size estimation and statistical analysis

Primary outcome of our study is the DASH-Score (0–100 points, 0 (no disability) to 100 (most severe disability)) [27]. In a case number calculation for unpaired samples and target figures we assume an effect size of a difference of 15 points at the highest as there is a standard deviation of 15 points as well. A difference of 15 points is seen as a minimal clinically important difference. This data has been published in other studies before. It has been tested for plausibility and has been taken over after that. Following parameters are the results: Delta = 15, SD = 15, alpha = 0.05, power = 0.8. *N* = 40 patients will be included according to the above mentioned calculation. To protect the quality of our study the drop-out rate should not exceed 20 % (e.g. 3 patients per group).

Standard deviations or confidence intervals, in the case of percentages, will be provided for each type of epidemiological data. The assumption of normality will be verified by the Shapiro-Wilk test for the use of parametric tests. A Pearson’s chi-square test will be employed to analyse results from the two groups involving categorical variables. A Student’s (parametric) *t*-test will be used for comparing groups of numeric variables. Paired t-tests (parametric) and Wilcoxon tests (non-parametric) will be used to compare clinical progression at follow-up intervals. The significance level used in all statistical tests is to be 5 % (alpha = 0.05), with tests having a *P* value less than 0.05 being statistically significant.

Should differences be found in primary outcomes, then statistical methods will be used to test whether there is robust correlation between epidemiological factors or fracture seriousness and the observed functional outcomes. In addition, we intend to employ Kaplan-Meier survival analysis to evaluate drop-outs should high rates of complications (greater than 20 %) occur in either assignment group.

Patients who experience treatment failures and require additional surgery will be monitored and their results computed in the primary assignment group (intention-to-treat principle). Provisions are to be made for blinded statistical analysis of data by a statistician who is unfamiliar with the objectives and outcomes of interest.

## Discussion

The present study protocol on the operative treatment of 2-part surgical neck fractures (AO 11-A3) of the proximal humerus in the elderly is the first study to compare a cement augmented locking plate versus a multiplanar proximal humerus nail. Both options might prevent secondary displacement after surgical treatment of 2-part-fractures.

Since secondary dislocation of the humeral head is still the most common complication after surgical treatment of proximal humeral fractures [10] various approaches exist to increase the biomechanical properties of the available constructs. One option is to increase the stability of the screw-bone-interface in locking plate devices by cement augmentation of the screw tips. Therefore cannulated screws are introduced in the humeral head and a small amount of Polymethylmethacrylat cement (PMMA) is injected around the screw tip. Various biomechanical trials demonstrate that the cement augmentation causes a noticeably higher primary stability. Unger et al. showed a significantly higher tolerance concerning varus-bending and axial-rotation after augmentation of the four most proximal screws [13]. Roderer et al. also achieved almost the same results concerning primary stability of the fracture by positioning of only two augmented screws in the region of the least bone density [15, 28]. Krappinger et al. recommended screw tip augmentation after studying predictors for failure of surgical fixation in proximal humerus fractures. They especially pointed out a reduced bone density as a main risk factor for failure of primary fixation [16]. Although screw tp augmentation is widely used, there is no study reporting on clinical results.

In a clinical retrospective study of 2- and 3-part-fractures in older patients (age: 60–83 years) Mihara et al. reported good results in terms of function, range of motion (ROM), pain and alignment of the former fracture elements after a mean follow-up of 14 months after treatment by nail [19]. A biomechanical trial by Furoria et al. resulted in higher rotational stability and tolerance against torsion after locking plate fixation compared to an intramedullary nail, whereas there was no significant difference in movement of the fracture elements [20]. Similar results were observed in a biomechanical study by Edwards et al. who found a significant stiffer bone-implant construct after usage of a proximal humeral nail compared to a locking compression plate [21]. Lekic et al. showed in a clinical study a comparable outcome after treatment of 2-part- fractures with intramedullary nail (IMN) and locking plate but assume a higher rate of complication after use of a nail [22]. A further development of IMNs is multiplanar nailing. Screws can be inserted in various different levels and directions (multiplanar) which can lead to less implant toggling and higher stability [23]. Using angle stable locking screws in the distal part of the humerus results in a significant lower movement of the fracture elements during rotational and bending load, especially in a postoperative early stage [24]. In a randomized controlled trial Zhu et al. compared the IMN with the non-augmented locking plate in 2-part surgical neck fractures of the proximal humerus and showed a lower complication rate in the IMN group whereas the outcome in the locking plate group was better. After three years no difference in the outcome measured by the ASES-Score was observed [25].

Respecting the increasing incidence of fragility fractures to the proximal humerus, there is an urgent need to optimize the stability of our fixation construct. With this RCT we aim to compare the clinical outcome of the latest implant designs addressing this problem.

## Abbreviations

ASES: American Shoulder and Elbow Score
CS: Constant Score
DASH: Disabilities of the Shoulder, Arm and Hand-Score
f: Female
m: Male
OSS: Oxford Shoulder Score
ROM: Range of motion
SF-36: Short Form 36
